# STDP induced synchrony in inhibitory neural networks: theory and experiments

**DOI:** 10.1186/1471-2202-13-S1-P32

**Published:** 2012-07-16

**Authors:** Zack Kagan, Charles J Frazier, Sachin S Talathi

**Affiliations:** 1 Department of Electrical and Computer Engineering, University of Florida, Gainesville, FL 32610, USA; 2 Department of Pharmacodynamics, University of Florida, Gainesville, FL 32610, USA; 3 Depatment of Pediatrics, University of Florida, Gainesville, FL 32610, USA; 4 Department of Biomedical Engineering, University of Florida, Gainesville, FL 32610, USA; 5 Dept of Neuroscience, University of Florida, Gainesville, FL 32610, USA

## 

Gamma rhythms have been the focus of significant research interest within the neuroscience community in recent years [1]. Earlier theoretical studies focused on the question: how can a network of GABA containing neurons generate gamma oscillations? The findings suggest that for non-instantaneous synaptic events, inhibition rather than excitation is a better synchronizing mechanism for the generation of gamma rhythms [2]. Synchrony in inhibitory neural networks however is extremely sensitive to intrinsic heterogeneity in the network [3]. Based on the evidence for spike timing dependent plasticity (STDP) in inhibitory neurons [4,5], we hypothesize that *STDP of inhibitory synapses can promote robust neural synchrony in inhibitory neuronal networks in the presence of heterogeneity*.

**Figure 1:**
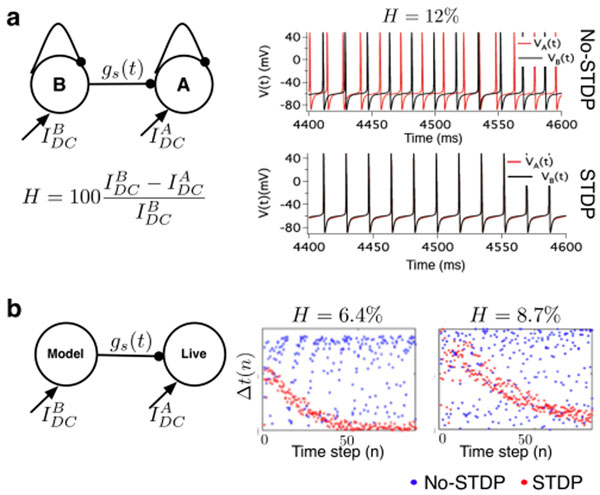
a) Theoretical model of UCI. Autaptic synapse is introduced to mimic spike frequency adaptation in neuronal firing activity. b) Hybrid UCI network. Dynamic clamp experimental results for experimental protocol with fixed static-synaptic strength g_s_(0)=0.5 mS/cm^2^.

We investigate our hypothesis in a computational (Figure 1a) and a hybrid (Figure 1b) uni-directionally coupled network (UCI) of two fast-spiking inhibitory neurons. In the computational model, each neuron is modeled using the Hodgkin-Huxley (HH) framework [4]. Heterogeneity (*H*) is modeled by the difference in the intrinsic firing activity of the coupled neurons resulting from different DC current input. In the presence of STDP, the strength of synaptic coupling between the coupled neurons is modeled using a linear additive rule as: *g_s_*(*t*) = *g_s_*(*t* – 1) + Δ*g*_STDP_(Δ*t*), where Δ*t* = *t*_A_ – *t*_B_ is the time interval between successive spikes of the post-synaptic and the pre-synaptic neurons and Δ*g*_STDP_ is the STDP rule adapted from [4]. In Figure 1a, we demonstrate the effect of STDP on synchrony between coupled neurons in the presence of and the absence of STDP for network heterogeneity H=12%. Theoretical analysis using the concept of spike time response curves and Arnold tongue revealed that STDP induced 1:1 synchrony in the UCI network is (i) robust against a large range of heterogeneity in the intrinsic firing activity of coupled neurons, and (ii) in-phase i.e., Δ*t* ≈ 0. Furthermore, the time to in-phase synchronization increases with increasing heterogeneity.

We used the dynamic clamp technique to construct a hybrid UCI network consisting of an HH based model of a inhibitory neuron coupled to a living inhibitory neuron in the stratum oriens of area CA1 of the hippocampus. Whole cell patch clamp recordings were obtained from the live cell under the following experimental protocols: (i) Fix *H*, vary the static synapse coupling strength *g_s_*(*0*) and (ii) Fix static synapse coupling strength *g_s_*(*0*) and vary *H*. In Figure 1b, we present results from the second experimental protocol. We see that as predicted by our theoretical model, the time to in phase neural synchrony in the hybrid UCI network increases with increasing heterogeneity.

We conclude that STDP of inhibitory synapses is a putative mechanism for robust neural synchrony in inhibitory neuronal networks.
